# The mediating role of social connectedness and hope in the relationship between group membership continuity and mental health problems in vulnerable young people

**DOI:** 10.1192/bjo.2023.500

**Published:** 2023-07-19

**Authors:** Claire Vella, Clio Berry, Matthew J. Easterbrook, Daniel Michelson, Leanne Bogen-Johnston, David Fowler

**Affiliations:** University of Sussex, UK; Brighton and Sussex Medical School, UK; King's College London, UK

**Keywords:** Group membership, social connectedness, hope, mental health, COVID-19 pandemic

## Abstract

**Background:**

There is growing evidence of a beneficial effect of social group processes on well-being and mental health.

**Aims:**

To investigate the role of group membership continuity in reducing mental ill-health among young people who were already vulnerable pre-pandemic, and to understand the social and psychological mechanisms of the benefits of group memberships for vulnerable young people.

**Method:**

This study takes a cross-sectional design, using survey data from a sample of 105 young people aged 16–35 years, collected approximately 1 year after the global COVID-19 outbreak (January to July 2021). Correlational and path analyses were used to test the associations between group membership continuity and mental health problems (depression, anxiety, psychotic-like experiences) and the mediation of these associations by hope and social connectedness (in-person and online). To correct for multiple testing, the Benjamini–Hochberg procedure was implemented for all analyses. Indirect effects were assessed with coverage of 99% confidence intervals.

**Results:**

Multiple prior group memberships were associated with preservation of group memberships during the COVID-19 pandemic. In-person social connectedness, online social connectedness and hope mediated the relationship between group membership continuity and mental health problem symptoms.

**Conclusions:**

The results suggest that clinical and public health practice should support vulnerable young people to foster and maintain their social group memberships, hopefulness and perceived sense of social connectedness as means of helping to prevent exacerbation of symptoms and promote recovery of mental health problems, particularly during significant life events.

In recent years, the prevalence of mental health problems in young adults has risen,^1^ with evidence that the COVID-19 pandemic has exacerbated this further.^2^ A call has been made for research to identify the mechanisms that underpin mental well-being,^3^ particularly for vulnerable young people and those with pre-existing mental health difficulties who were expected to be disproportionately affected by the pandemic.^4^

The social cure theory (also known as the social identity approach to health) is a promising theoretical perspective developed to bridge the social and psychological dimensions of health and well-being.^5^ This approach has two key features: the importance of social group processes in health, and the importance of people's psychological identification with social groups (i.e. their social identities: the extent to which people internalise their group memberships as a part of their sense of self^6^). Evidence suggests that identification with groups can affect how we feel, behave and interact with others.^7^ In turn, group social identities provide psychological resources, such as a sense of connection, meaning, support and personal control, which support health and well-being.^7,8^

## Group membership processes and mental health problems

A key hypothesis in the social cure agenda is that the more subjectively important social identities an individual has, the greater the benefit to their health (the multiple identities hypothesis).^8^ In support of this theory, having fewer group memberships has been found to be associated with elevated symptoms of depression in vulnerable young people^9^ and with the presence of psychosis in a clinical sample.^10^ In adults, identification with multiple groups is considered to be a better predictor of well-being than frequency of social contact^11^ and is associated with lower self-rated depression, lower odds of being given an antidepressant prescription^12^ and reduced risk of relapse.^13^

The protective effect of multiple group memberships is also observable in the context of significant life events and life transitions. Belonging to multiple group memberships before a life event is related to a higher likelihood of preserving group memberships after the life event.^14^ Haslam and colleagues describe this as ‘having one's eggs in multiple baskets enhances the likelihood of having some of those eggs intact after an accident’ (p. 675).^15^ In the context of life transitions, the maintenance of multiple group memberships is associated with greater life satisfaction, fewer symptoms of depression and lower levels of perceived stress.^16^ In adult and student samples, emerging evidence from the COVID-19 pandemic (as a significant life event) is also beginning to show an effect of group membership and identity continuity that protects against loneliness and promotes well-being and mental health during periods of lockdown and enforced social restrictions.^17–19^

## Social connectedness and hope as mechanisms linking group membership and mental health problems

One of the main psychological resources hypothesised to be afforded by group social identities is a subjective sense of connection.^8^ Theories suggest that this may be because of the opportunities groups can offer for interaction and support, as well as our intrinsic drive to perceive similarities and relatedness with other group members.^20^ Although increased social connectedness is consistently associated with improvements in well-being and mental health symptoms in older adolescents^21^ and adults,^22^ only a small body of research has begun to investigate and provide evidence for the mediating role of a sense of connection in the relationship between group membership and health and well-being.^11,23,24^ There is also little (and sometimes contradictory) evidence around the impact of online social connection compared with in-person social connection. Research shows that increased text-messaging, online communication and social media use is associated with poorer mental and psychosocial health.^25,26^ Other research, however, indicates that online social connection and identity is associated with lower loneliness^27^ and increased self-esteem.^28^

Another psychological resource that has received little attention in the social cure literature is hope, a trait that represents an individual's perceived ability to achieve and pursue a desired goal.^29^ An early theory suggests that group processes can enable people to gain higher levels of hope, as groups can identify and influence a person's values, roles and plans for how activities could be achieved.^30^ Previous research has shown that hope can be strengthened through positive relationships and can function as a mediator in the relationship between social support and mental-health-related outcomes.^29^ Hopefulness is associated with improved mental health, including reduced depression^29^ and anxiety,^31^ whereas hopelessness is positively associated with significant outcomes such as suicidal ideation and suicide attempts.^32^ Hopefulness is also considered to promote perceived emotional control and well-being during adverse life events, such as the COVID-19 pandemic.^33^ Further investigation of hope as a beneficial psychological resource afforded by social identity processes would offer invaluable evidence about how vulnerable individuals and care providers could harness a sense of hopefulness. To date, only a few studies have investigated the association of group identification and multiple group memberships with measures of personal control and volitional agency;^24^ however, most studies have promisingly observed a significant association with well-being.^23,34,35^

## Current study

Most prior research has examined the health impact of social group memberships in non-clinical adult samples or university students, often with a narrow focus on depression or life satisfaction. Relatively little is known about the application of the social cure approach to other mental health problems, such as anxiety and psychotic-like experiences, which are commonly comorbid with depression in young people.^36,37^ There are preliminary indications from clinical samples that social identity processes are associated with improvements in depression,^18^ addiction and well-being.^7,16^ Further research is still needed to understand the clinical utility of the social cure approach across diverse and complex samples, contexts and mental health problems. It is important to address the implications for targeting social group memberships in vulnerable and psychiatric samples, as such populations, in comparison with the general population, can experience significant difficulties with forming and maintaining social groups and are at heightened risk of belonging to groups that can perpetuate a stigmatised identity.^38^ As the COVID-19 pandemic forced people to increasingly rely on digital means to communicate and remain connected with social groups,^39^ it is therefore also important to investigate the clinical application of the social cure approach within this unique social context.

This study is among the first to investigate the associations of group membership continuity with vulnerable young people's symptoms of depression, anxiety and psychotic-like experiences, at an under-investigated time-point approximately 1 year into the COVID-19 pandemic. It is also among the first to investigate the separate influences of in-person social connectedness, online social connectedness and hope as potential mediators of these associations. Collectively, this investigation extends our knowledge about the context and possible mechanisms through which the social cure approach may support diverse mental health difficulties in young and vulnerable populations. It also provides invaluable insights into how to support such populations through future global health crises, adverse life events and post-pandemic recovery.

This study tests the hypotheses that a greater perceived number of multiple group memberships before the COVID-19 pandemic is associated with greater membership continuity during the COVID-19 pandemic (H1), and greater membership continuity is associated with fewer symptoms of depression, anxiety and psychotic-like experiences (H2). We also consider the hypotheses that greater social connectedness (in-person and online) and hope is associated with fewer symptoms of depression, anxiety and psychotic-like experiences during the COVID-19 pandemic (H3), and these psychological resources mediate the relationship between membership continuity and symptoms of mental health problems (H4).

## Methods

### Design

The present study has a cross-sectional observational design using baseline survey data collected from January 2021 to July 2021 as part of the DisCOVery study, a longitudinal mixed-methods study investigating the social and mental health impacts of the COVID-19 pandemic on vulnerable young people^a^.

### Participants

The study operationalised ‘vulnerable’ young people as those who were experiencing social and mental health difficulties and/or were accessing statutory or third-sector youth organisations and living in areas of socioeconomic deprivation, including rural and coastal communities. In total, 105 young people aged 16–35 years were recruited from Sussex, Kent, Surrey, Norfolk and Suffolk (UK). Participants were eligible for the DisCOVery study if they were in contact with mental health, social care or voluntary-sector services or self-identified as experiencing mental health problems. From the 105 eligible cases, 25 young people (23.81%) considered to have complex emerging mental health problems and social disability were recruited through their previous involvement in the PRODIGY trial.^40^

### Measures

#### Group membership

Multiple group memberships before the COVID-19 pandemic (prior multiple memberships) and the maintenance of group memberships during the COVID-19 pandemic (membership continuity) were measured separately using four-item scales from the Exeter Identity Transition Scale (EXITS).^15^ All items were self-reported on a seven-point Likert scale, ranging from 1 = strongly disagree to 7 = strongly agree. An example item of prior multiple memberships is ‘Before COVID-19, I belonged to many different groups’. An example item of membership continuity is ‘Since COVID-19, I still belong to the same groups I was a member of before COVID-19’. Higher scores reflected higher levels of prior multiple group memberships or membership continuity. For each dimension, scores ranged from 4 to 21. The total score of each four-item scale was used (prior multiple memberships α = 0.94; membership continuity α = 0.89).

#### Depression

Symptoms of depression were measured using the Patient Health Questionnaire – Depression Scale (PHQ-9).^41^ Nine items were rated on a four-point Likert scale, for example, ‘Over the last 2 weeks, how often have you been bothered by any of the following problems? Little interest or pleasure in doing things’ (0 = not at all, 1 = several days, 2 = more than half the days, 3 = nearly every day). Higher scores indicated higher levels of depressive symptoms, categorised as minimal = 0–4, mild = 5–9, moderate = 10–14, moderately severe = 15–19 and severe = 20–27. The total score for all items was used (α = 0.90).

#### Anxiety

Symptoms of anxiety were measured using the Generalised Anxiety Disorder-7 *(*GAD-7).^42^ Seven items were rated on a four-point Likert scale, for example ‘Over the last 2 weeks, how often have you been bothered by the following problems? Feeling nervous, anxious or on edge’ (0 = not at all, 1 = several days, 2 = more than half the days, 3 = nearly every day). Higher scores indicated higher levels of anxiety symptoms, categorised as minimal = 0–4, mild = 5–9, moderate = 10–14 and severe = 15–21. The total score for all items was used (α = 0.93).

#### Psychotic-like experiences

Psychotic-like experiences were measured using the Community Assessment of Psychic Experiences (CAPE-P15).^43^ Fifteen items were rated on a four-point Likert scale, for example ‘In the past three months have you … seen objects, people or animals that other people can't see’ (0 = never, 1 = sometimes, 2 = often, 3 = nearly always). Higher scores indicated higher levels of psychotic-like experiences. The total score for all items was used (α = 0.94).

#### In-person and online social connectedness

In-person and online social connectedness was measured using the Social Connectedness Scale.^44^ Eight items were rated on a six-point Likert scale, for example ‘I feel so distant from people’ (1 = strongly agree to 6 = strongly disagree). Scores ranged from 8 to 48, with higher scores indicating higher levels of social connectedness. Participants were asked to complete the measure twice, first thinking about in-person interactions and relationships, and then thinking of online interactions and relationships. The total score for all items was used (in-person social connectedness α = 0.94; online social connectedness α = 0.95).

#### Hope

Levels of hope were measured using the Trait Hope Scale.^45^ Twelve items were rated on an eight-point Likert scale, for example ‘I can think of many ways to get out of a jam’ (1 = definitely false to 8 = definitely true). A total hope score was computed by summing eight of the scale items, removing the four ‘distractor’ items. Scores ranged from 8 to 64, with higher scores indicating higher levels of hope. The total score was used (α = 0.90).

#### Online and in-person social group interaction

Two items adapted from the EXITS^7,15^ were used to measure the number of times participants interacted with their social groups over the previous week. Online interaction was captured by the question: ‘In the last week, how many times have you interacted with your groups online?’. In-person interaction was captured by the question: ‘In the last week, how many times have you interacted face-to-face (in-person, not online) with your groups?’.

### Procedure

Mental health, community and social care services in the relevant geographical locations were asked to advertise the study poster via social media and to discuss the opportunity with the young people accessing their service. Individuals previously involved in the PRODIGY research trial were invited to take part by the research team via email or telephone. Data were collected via an online survey, web-hosted by Qualtrics. The survey took approximately 45 min to complete and included a battery of self-administered questionnaires that focused on COVID-19 concerns, social functioning and mental health problems. Participants without access to the internet, or those who required support, were invited to complete the survey with a member of the research team via telephone. Ethical approval for the study was provided by the Health Research Authority (HRA), Essex Research Ethics Committee (reference 20/EE/0238). Participants provided electronic written informed consent before taking part. Following HRA regulations, young people aged 16 and over provided consent on their own behalf.

### Data analysis

Hypotheses were tested using bivariate correlations in IBM SPSS Statistics (version 27) and simple mediation analysis in Hayes PROCESS version 4 for SPSS^46^ with ordinary least squares regression models (see Supplementary Appendix 1 available at https://doi.org/10.1192/bjo.2023.500 for power calculation information). Effect sizes for bivariate correlations were interpreted using Cohen's operational guidelines.^47^ To correct for multiple testing, the Benjamini–Hochberg procedure^48^ was implemented on grouped analyses: (a) missing data checks, (b) bivariate correlations, (c) mediation models predicting depression, (d) mediation models predicting anxiety and (e) mediation models predicting psychotic-like experiences. To conservatively assess the significance of indirect effects in the context of multiple directed-path models, confidence intervals with 99% coverage were implemented. Sensitivity analyses retested each model, adjusting separately for age, gender and ethnicity. To control for current mental health problems being a function of long-lasting pre-existing mental health problems, each model was retested adjusting for years since mental health difficulties started. Furthermore, mental health problems are predicted differently by social contact versus social identification.^11^ To control for the relationship between membership continuity and mental health problems being a function of the number of social interactions – rather than the subjective sense of social connectedness – each model was retested adjusting separately for the number of in-person interactions and number of online interactions with social groups. Following data screening (Supplementary Appendix 1), robust confidence intervals and standard errors were computed for all applicable analyses.

## Results

### Sample characteristics

Sample characteristics are presented in Table 1. Participants were mainly female (*n* = 75, 71.4%) and White British/White other (*n* = 93, 88.6%), with a mean age of 24 years (s.d. = 3.6). Most participants were either studying, in training or employed (*n* = 60, 59%), but a large proportion were not in employment, education or training (35.2%). Most participants reported having a disability, long-term illness or health condition (*n* = 67, 65%) and pre-existing mental health difficulties (*n* = 98, 94.2%). On average, their mental health problems had started 9.38 years ago (s.d. = 5.48), and at least 64.76% (*n* = 68) of the sample reported two or more diagnoses. Participants interacted with their social groups on average 1.48 times in person and 3.4 times online per week. For in-person interaction, 77.3% (*n* = 75) of participants reported that they had less interaction than before the COVID-19 pandemic, and 5.2% (*n* = 5) reported more than before. For online interaction, 44.7% (*n* = 42) reported less interaction than before and 21.3% (*n* = 20) as more than before.

**Table 1:** Sample characteristics

	*N* (%)	Mean (s.d.)	Range
Age, years	(*n* = 105)	24 (3.6)	16–35
Social group interaction			
Number of online interactions	(*n* = 99)	3.40 (4.26)	0–20
Number of in-person interactions	(*n* = 97)	1.48 (3.01)	0–25
Gender	(*n* = 105)		
Female	75 (71.4)		
Male	25 (23.8)		
Non-binary/other	5 (4.8)		
Ethnicity	(*n* = 105)		
White British/White other	93 (88.6)		
Mixed ethnicity	7 (6.7)		
Asian British/Asian other	3 (2.9)		
Black British/Black other	2 (1.9)		
Employment status	(*n* = 103)		
NEET	37 (35.2)		
Furloughed	3 (2.9)		
Student (in college or university)	23 (23.8)		
Employed (part or full time)	13 (12.4)		
Gained new employment	14 (13.3)		
Enrolled in new training or study	10 (9.5)		
Homemaker/full-time parent	3 (2.9)		
Living situation	(*n* = 105)		
Live alone	17 (16.2)		
Friends/flatmates	8 (7.6)		
Partner/spouse	19 (18.1)		
Family	50 (47.6)		
Group accommodation	6 (5.7)		
Homeless	1 (1)		
Other	4 (3.8)		
Pre-existing mental health problems	(*n* = 104)		
Yes – received professional diagnosis	92 (88.5)		
Yes – not received professional diagnosis	6 (5.8)		
No	6 (5.8)		
Disability, long-term illness or health condition	(*n* = 103)		
Yes	67 (65)		
No	36 (35)		

NEET, not in employment, education or training.

The descriptive statistics for each study variable are shown in Table 2. Across the sample, 50.5% (*n* = 50) of participants scored above the PHQ-9 threshold warranting treatment for depression (moderately severe to severe symptoms), with 8.1% (*n* = 8) below the threshold for needing any treatment (minimal depression). Similarly, using the GAD-7 thresholds, 40% (*n* = 40) of the sample were experiencing severe symptoms of anxiety and 16% (*n* = 16) were experiencing minimal symptoms. Using a cut-off mean value of 1.47,^49^ 14.4% (*n* = 14) of the sample scored above the CAPE-15 threshold for ultra-high risk for psychosis.

**Table 2:** Descriptive statistics and bivariate correlations for hypotheses 1, 2 and 3

Variable	*N*	Mean (s.d.)	Range	Bivariate correlations							
				1	2	3	4	5	6	7	8
1. PHQ-9	99	15.12 (7.47)	0–27	1							
2. GAD-7	100	11.90 (6.49)	0–21	0.79***	1						
3. CAPE-P15	97	10.74 (10.24)	0–45	0.56***	0.52***	1					
4. In-person SC	100	23.91 (10.77)	8–48	−0.62***	−0.55***	−0.37***	1				
5. Online SC	99	24.99 (10.89)	8–48	−0.49***	−0.49***	−0.30**	0.77***	1			
6. PMM	100	15.67 (7.07)	4–28	−0.15	−0.13	−0.15	0.21*	0.12	1		
7. MC	101	13.41 (5.96)	4–28	−0.31**	−0.34***	−0.30**	0.38***	0.29**	0.24*	1	
8. TH	98	34.20 (13.01)	8–59	−0.49***	−0.34***	−0.41***	0.48***	0.45***	0.47***	0.48***	1

SC, social connectedness; PMM, prior multiple group memberships; MC, membership continuity; TH, trait hope.

*P < 0.05, **P < 0.01, ***P < 0.001.

### Associations between prior multiple group memberships, membership continuity, social connectedness, hope and symptoms of mental health problems (H1–H3)

Bivariate correlations testing the association between each study variable are presented in Table 2. All significant associations were robust to correction for multiple testing.

Having multiple prior group memberships before the COVID-19 pandemic was significantly positively related to membership continuity during the pandemic (95% BCa CI^b^ [0.01, 0.45]), representing a small to medium effect size. Membership continuity during the COVID-19 pandemic was significantly negatively correlated with symptoms of depression (95% BCa CI [−0.49, −0.10]), anxiety (95% BCa CI [−0.52, −0.13]) and psychotic-like experiences (95% BCa CI [−0.48, −0.11]), each representing a medium effect.

In-person social connectedness was significantly negatively correlated with symptoms of depression (95% BCa CI [−0.76, −0.47]) and anxiety (95% BCa CI [−0.70, −0.37]) with a large effect size, and with psychotic-like experiences (95% BCa CI [−0.55, −0.18]) with a medium effect size. Online social connectedness was significantly negatively correlated with symptoms of depression (95% BCa CI [−0.65, −0.30]) and anxiety (95% BCa CI [−0.64, −0.29]) with a medium to large effect size, and with psychotic-like experiences (95% BCa CI [−0.50, −0.06]) with a medium effect size. Hope was significantly negatively correlated with symptoms of depression (95% BCa CI [−0.67, −0.31]) and psychotic-like experiences (95% BCa CI [−0.56, −0.24]) with a medium to large effect size, and with anxiety (95% BCa CI [−0.53, −0.15]) with a medium effect size.

### *Post hoc* exploratory analysis

Exploratory partial correlations showed that relationships of symptoms of depression, anxiety and psychotic-like experiences with in-person social connectedness remained significant after controlling for online social connectedness. The relationships with online social connectedness, however, did not remain significant after controlling for in-person social connectedness (Supplementary Appendix 1).

### Social connectedness and hope as mediators in the relationship between group membership continuity and symptoms of mental health problems (H4)

**Fig. 1:**
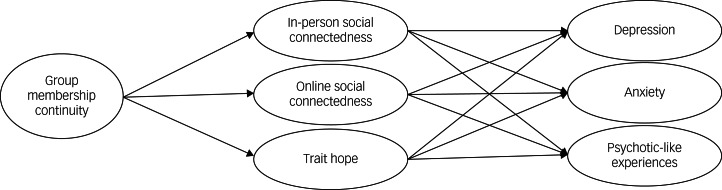
Conceptual model of hypothesis 4: group membership continuity as a predictor of symptoms of mental health problems, mediated by social connectedness (in-person and online) and hope.

In-person social connectedness, online social connectedness and hope as mediators of the relationships between membership continuity and symptoms of depression, anxiety and psychotic-like experiences during the COVID-19 pandemic were assessed using nine simple mediation models (Fig. 1 and Table 3). Correcting for multiple testing yielded no changes to the level of significance for each model parameter.

**Table 3:** Simple mediation path models testing the indirect effects of in-person social connectedness, online social connectedness and hope in the relationships between membership continuity and symptoms of mental health problems

Mediator variable/Path	Depression (PHQ-9)					Anxiety (GAD-7)					Psychotic-like experiences (CAPE-P15)				
	B (β)	SE	*t*(d.f.)	99% CI	*R*^2^	B (β)	s.e.	*t*(d.f.)	99% CI	*R*^2^	B (β)	s.e.	*t*(d.f.)	99% CI	*R*^2^
Mediator variable: in-person SC															
Path *a*	0.72 (0.39)***	0.18	4.10(95)	[0.19, 1.15]	0.15	0.69 (0.38)***	0.19	3.99(96)	[0.18, 1.15]	0.14	0.70 (0.38)***	0.18	3.99(95)	[0.20, 1.17]	0.14
Path *b*	−0.41 (−0.60)***	0.07	−6.92(94)	[−0.60, −0.22]	0.40	−0.30 (−0.50)***	0.06	−5.46(95)	[−0.45, −0.14]	0.32	−0.29 (−0.31)**e	0.13	−2.99(94)	[−0.61, 0.03]	0.16
Path *c′* (direct effect)	−0.09 (−0.07)	0.14	−0.79(94)	[−0.48, 0.26]		−0.16 (−0.14)	0.13	−1.55(95)	[−0.50, 0.16]		−0.29 (−0.17)	0.23	−1.65(94)	[−0.89, 0.27]	
Path *c* (total effect)	−0.38 (−0.30)**	0.12^b^	−3.09(95)	[−0.70, −0.06]^b^	0.09	−0.37 (−0.33)***^f^	0.11^a^	−3.42(96)	[−0.65, −0.08]^b^	0.11	−0.49 (−0.28)**^g,h^	0.17^a^	−2.89(95)	[−0.95, −0.04]^b^	0.08
	Indirect effect:					Indirect effect:					Indirect effect:				
Path *ab*	−0.29 (−0.23)	0.10	−	**[−0.59, −0.07]**		−0.21 (−0.19)	0.08	−	**[−0.44, −0.05]**		−0.20 (−0.12)	0.12	−	[−0.59, 0.01]^c^	
Mediator variable: online SC															
Path *a*	0.55 (0.29)**^i^	0.19	2.97(94)	[0.07, 1.01]	0.09	0.48 (0.26)*	0.19	2.60(95)	[−0.00, 0.95]	0.07	0.48 (0.26)*	0.19	2.60(94)	[−0.01, 0.96]	0.07
Path *b*	−0.31 (−0.46)***	0.07	−5.07(93)	[−0.48, −0.14]	0.29	−0.25 (−0.42)***	0.06	−4.65(94)	[−0.39, −0.10]	0.27	−0.24 (−0.25)*^j^	0.12	−2.51(94)	[−0.54, 0.06]	0.14
Path *c′* (direct effect)	−0.21 (−0.17)^e^	0.14	−1.83(93)	[−0.60, 0.16]		−0.24 (−0.22)*	0.12	−2.42(94)	[−0.56, 0.06]		−0.38 (−0.22)*^k^	0.20	−2.21(93)	[−0.89, 0.12]	
Path *c* (total effect)	−0.37 (−0.30)**	0.12^a^	−3.08(94)	[−0.70, −0.06]^b^	0.09	−0.36 (−0.33)**	0.11^a^	−3.40(95)	[−0.65, −0.08]^b^	0.11	−0.50 (−0.28)**	0.17^a^	−2.89(94)	[−0.95, −0.04]^b^	0.08
	Indirect effect:					Indirect effect:					Indirect effect:				
Path *ab*	−0.17 (−0.14)	0.08	−	**[−0.40, −0.02]**^m^		−0.12 (−0.11)	0.06	−	[−0.30, 0.00]^d^		−0.11 (−0.06)	0.09	−	[−0.42, 0.03]	
Mediator variable: trait hope															
Path *a*	1.02 (0.46)***	0.21	5.02(94)	[0.49, 1.56]	0.21	1.06 (0.48)***	0.20	5.29(95)	[0.49, 1.54]	0.23	1.02 (0.46)***	0.21	5.02(94)	[0.46, 1.54]	0.21
Path *b*	−0.27 (−0.47)***	0.06	−4.69(93)	[−0.41, −0.11]	0.26	−0.12 (−0.25)*^j^	0.05	−2.33(94)	[−0.26, 0.02]	0.15	−0.28 (−0.36)**^l,e^	0.08	−3.38(93)	[−0.50, −0.06]	0.18
Path *c′* (direct effect)	−0.09 (−0.07)	0.14	−0.68(93)	[−0.45, 0.26]		−0.21 (−0.19)^e^	0.12	−1.79(94)	[−0.55, 0.10]		−0.21 (−0.12)	0.19	−1.11(93)	[−0.69, 0.29]	
Path *c* (total effect)	−0.36 (−0.29)**^h^	0.12^a^	−2.89(94)	[−0.68, −0.03]^b^	0.08	−0.34 (−0.31)**	0.11^a^	−3.22(95)	[−0.62, −0.06]^b^	0.10	−0.50 (−0.28)**^g,i^	0.17^a^	−2.84(94)	[−0.96, −0.04]^b^	0.08
	Indirect effect:					Indirect effect:					Indirect effect:				
Path *ab*	−0.27 (−0.22)	0.07	−	**[−0.50, −0.10]**		−0.13 (−0.12)	0.06	−	[−0.30, 0.03]^c^		−0.29 (−0.16)	0.10	−	**[−0.58, −0.07]**	

Confidence intervals and standard errors are based on 5000 bootstrap samples.

SC, social connectedness; B, unstandardised coefficient; *β*, standardised coefficient.

a. Non-bootstrapped standard error.

b. Non-bootstrapped 99% confidence intervals.

c. Indirect effect present in simple mediation model and each covariate model with 95% confidence intervals, excluding model controlling for years since mental health difficulties started.

d. Indirect effect present in simple mediation model and each covariate model with 95% confidence intervals.

e. Significant at *P* < 0.05 in models controlling for years since mental health difficulties started.

f. Significant at *P* < 0.01 level in models controlling for gender, years since mental health difficulties started, in-person contact and online contact.

g. Significant at *P* < 0.05 in models controlling for gender.

h. Significant at *P* < 0.05 in models controlling for in-person contact.

i. Significant at *P* < 0.05 in models controlling for online contact.

j. Non-significant in models controlling for years since mental health difficulties started.

k. Non-significant in models controlling for gender.

l. Significant at *P* < 0.001 in models controlling for age, gender and online contact.

m. Indirect effect robust to each covariate in models with 95% confidence intervals.

*P < 0.05, **P < 0.01, ***P < 0.001.

There were significant indirect effects via in-person social connectedness of membership continuity on symptoms of depression and of membership continuity on symptoms of anxiety. However, there was a non-significant indirect effect via in-person social connectedness of membership continuity on psychotic-like experiences. For these models, the direct effects of membership continuity on depression and on anxiety were not significant, whereas the total effects were significant.

There was a significant indirect effect via online social connectedness of membership continuity on symptoms of depression. For this model, the direct effect was not significant, whereas the total effect was significant. There were non-significant indirect effects via online social connectedness of membership continuity on symptoms of anxiety, and of membership continuity on psychotic-like experiences. For these models, the direct and total effects of membership continuity on anxiety and on psychotic-like experiences were significant.

There were significant indirect effects via hope of membership continuity on symptoms of depression and on psychotic-like experiences. However, there was a non-significant indirect effect via hope of membership continuity on symptoms of anxiety. For these models, the direct effects of membership continuity on depression, psychotic-like experiences and anxiety were not significant, whereas the total effects were significant.

### Sensitivity analyses

The significant indirect effects via in-person social connectedness and hope were robust (with 99% confidence intervals) to controlling for age, gender, years since mental health difficulties started, and both the number of in-person interactions and the number of online interactions with social groups (indirect effects ranged from −0.17 to −0.34, bootstrapped 99% CI ranged from −0.66 to −0.01). The significant indirect effect via online social connectedness was only robust with 99% confidence intervals to controlling for age (indirect effect = −0.17, bootstrapped 99% CI [−0.41, −0.01]) and was rendered non-significant after adjustments for gender, years since mental health difficulties started, and numbers of in-person and online interactions with social groups (indirect effects ranged from −0.13 to −0.17, bootstrapped 99% CI ranged from −0.34 to 0.02).

## Discussion

This cross-sectional study is among the first to investigate the relationships between self-reported group membership, in-person social connectedness, online social connectedness and hope with respect to vulnerable young people's self-rated symptoms of depression, anxiety and psychotic-like experiences. It is also among the first to investigate the relevance of the social identity approach to health for vulnerable young people during a period of longer-term adjustment to the ongoing and evolving lockdown and social distancing measures, approximately 1 year into the COVID-19 pandemic (January 2021 to July 2021). Collectively, this study aimed to add novel evidence around how, when, for whom and for which mental health problems group memberships may have a protective effect.

As hypothesised, having a greater perceived number of multiple group memberships before the COVID-19 pandemic was significantly associated with greater membership continuity during the pandemic. Higher levels of membership continuity during the pandemic were significantly bivariately associated with higher scores of in-person social connectedness, online social connectedness and hope. Higher levels of membership continuity, in-person social connectedness, online social connectedness and hope were also all associated with lower scores for depression, anxiety and psychotic-like experiences. These results support the existing theoretical and empirical literature that detail the social identity approach to health^5^ and the beneficial role of preserved group memberships during significant life events.^16^ They also add to the growing body of literature that recognises the importance of social groups and identity continuity for aspects of well-being during the COVID-19 pandemic.^17–19^ Extending past literature, however, they offer some of the first evidence towards the significance of group membership preservation with respect to reduced mental health problems in a sample of vulnerable young people who had experienced approximately a year or more of global pandemic risks and restrictions that were not common to most stressors or adverse life events.

This study also provides novel evidence for mechanisms that may underpin the beneficial effects of preservation of group memberships for vulnerable young people. As hypothesised, membership continuity was significantly associated with fewer symptoms of depression through mediating effects of greater hope, and with both in-person and online social connectedness. The significance of all three mechanisms fits with the theory that the psychological resources afforded by multiple group memberships can be understood as a ‘suite’ of psychological needs and should not be in competition as the one ‘true’ mechanism.^7,23^ These findings also support the concept of social connectedness and hope as promising mechanisms in the relationship between group membership and self-rated mental health problems. Specifically, the results support the connection hypothesis and agency hypothesis,^7,8,34^ as well as recent evidence for the beneficial role of hopefulness and social connectedness in greater well-being during the COVID-19 pandemic.^18,33^

This being said, the association between membership continuity and reduced anxiety was only significantly mediated through in-person social connectedness, and the association with reduced psychotic-like experiences was only significantly mediated through hope. Moreover, although each of the mediating effects of hope and in-person social connectedness remained after controlling for age, gender, years since mental health difficulties started, and numbers of in-person and online interactions with social groups, the mediating effect of online social connectedness only remained after controlling for age. These findings suggest that the protective effects of membership continuity against different mental health problems may have different underlying mechanisms, at least for vulnerable young people during the COVID-19 pandemic. This is supported by a recent suggestion that a nuanced application of the social identity approach to health may be needed for diagnoses such as psychosis.^38^

One explanation for why hope was the only significant mediator between membership continuity and reduced psychotic-like experiences is that the related concepts of self-esteem and locus of control have long been established as key risk and recovery factors for psychosis. Consequently, it has been proposed that a lack of social identity perpetuates low self-esteem and an external locus of control in migrant populations with elevated rates of psychosis.^50^ Qualitative data from individuals experiencing symptoms of psychosis also describe how friends are seen as a source of hope.^51^ However, a sense of connection to others does not always have a beneficial impact on well-being, as it can perpetuate the personal identity as someone who is ‘ill’ with a highly stigmatised condition.^38^ The finding that membership continuity was associated with anxiety only through the indirect effect of in-person social connection is less easily explained. Although this broadly aligns with the evidence that in-person social support – and not online social support – is associated with reduced anxiety,^52^ it contests the evidence that hopefulness is consistently associated with lower anxiety.^31^ As hope is relationally driven, it is possible that social groups can negatively affect levels of hope. However, more research is clearly needed to understand how and why group processes and the potential psychological resources they reinforce may be more or less strongly associated with different mental disorders or symptom profiles.

Last, this study reveals a notable difference in the mediating role of social connectedness. For this sample of vulnerable young people, a more robust mediation effect was observed for in-person social connectedness than online social connectedness in the relationship between membership continuity and symptoms of depression and anxiety. This suggests that a sense of connection from in-person interactions and relationships may be a more important mechanism underlying the benefits of membership continuity for health. This supports recent research that found that online interaction with others may be inadequate compared with in-person contact.^53^ For instance, during the pandemic, increased time connecting to friends virtually was associated with greater depression,^26^ and online social connections only protected well-being under the most restrictive stay-at-home measures.^39^ This follows evidence that although online contact may promote the formation of online groups and communities, it can also create a source of alienation and ostracism.^53^ This is explained by the interpersonal-connection-behaviours framework.^54^ The impact of online social interaction may depend on how people interact online (including social media use versus technology-mediated communication), the purpose of the interaction and the extent to which online interaction is relied upon. As the data for this study were collected over months of loosening social distancing restrictions, the results may reflect the fact online social connection had less of a beneficial impact on mental health as opportunities for in-person social connection were increasingly permitted.

### Limitations and future directions

The cross-sectional nature of the research meant that causal relationships could not be directly tested. It could be argued that those who were more resilient or able to maintain better mental health were better able to maintain their social group memberships.^55^ This would mean that group processes, social connection and hope were not protective mechanisms leading to reduced mental health problems during the COVID-19 pandemic but were consequences for those who, for another reason, experienced fewer mental health problems. The direction of the results of this study are supported by past strong evidence that numbers of social groups and levels of loneliness or social connection predict changes in mental health over time, and not *vice versa*.^11,56^ Nevertheless, future research should aim to test the presented associations using longitudinal data and test the potential mechanisms as a part of a causal framework within a social groups intervention study.

There are also some important limitations of the measurement approach used in the present study. The data were collected over a period that saw gradual easing of lockdown and social distancing measures. It is not clear how this easing, which would have allowed more in-person social contact and the possibility of returning to pre-pandemic daily activities, may have affected the results. Multiple group memberships before the COVID-19 pandemic were self-reported retrospectively, with no independent way to corroborate the data collected. As this study was conducted approximately 1 year after the outbreak of the pandemic, participants’ perceptions of their social world a year earlier may have been inaccurate. Moreover, understanding what constitutes a ‘group’ and what constitutes belonging to ‘lots’ of groups can be quite nuanced and subjective. Unlike previous research,^12,13,34,55^ this study did not restrict data collection to a set number of groups or a list of predetermined types of groups. However, no explicit information about the number or type of groups that participants felt they were a member of was collected. The extent to which participants’ in-person and online social interactions involved the same or different social groups is also unknown. Similarly, this study did not collect information about the level of identification with each group membership, which the social identity approach suggests is associated with the impact of being a member of that group.^13^ This means that this study does not provide rich detail about the number or nature of groups or the level of identification with those groups, which may be particularly beneficial to vulnerable young people's mental health during the COVID-19 pandemic or similar adverse life events. Future research should collect explicit data about numbers and types of groups, as well as levels of identification with both in-person and online groups, and how these relate to different mental health problems for young people. Such data could be measured via social identity mapping, a tool developed to represent an individual's multidimensional network of social identities.^57,58^

It should also be noted that this study used a measure of trait hope, rather than a state-based measure of hope. This could be considered a limitation as it did not account for how levels of hope at the time of the study may have been situational in the context of the COVID-19 pandemic. Trait hope is likely to be strongly correlated with state-based hope, and it is possible that a more robust indirect effect on state-based hope would have been observed. Nevertheless, previous research has typically used more dispositional measures of personal control. Moreover, stable and persistent hopelessness, rather than situational hopelessness, has been associated with a higher risk of mental health difficulties.^32^ Therefore, the inclusion of trait hope in this study was considered a more appropriate and conservative measure. Further research could investigate whether hopefulness, as afforded by group social identities, should be understood as a fluid psychological process or as a sustained resource that may act as a psychological buffer for ongoing and future significant life events.

Finally, the relatively small sample size and preponderance of White British females could affect the interpretation and generalisability of the results. Recent evidence has shown that differences in race, ethnicity and gender were associated with differences in well-being and social disconnection during the pandemic.^59,60^ This means the findings reported in this study may be particularly indicative of those who identify as a woman, White and/or British.

### Implications

The present findings help to increase our understanding about ways to protect and support vulnerable young people through global health crises and adverse life events. They suggest that young people should be supported to foster and maintain their social group memberships, hopefulness and perceived sense of social connectedness to prevent and support recovery from mental health problems. This supports the growing body of literature that calls for researchers, commissioners, policy makers and governments to seriously acknowledge the social determinants of health and the emerging importance of social-based interventions for young people's mental health. Social-identity-based innovations with the potential to be integrated into services and educational settings include the online group maintenance activity Groups 2 Connect^61^ and the short, theory-driven, evidence-based social group intervention Groups 4 Health.^10^ Practitioners, teachers and family members supporting vulnerable young people could also aim to provide regular opportunities to remain connected with others who share similar lived experiences or interests.

In summary, this study found evidence that multiple group memberships were associated with the preservation of group memberships during the COVID-19 pandemic, and that preserved group memberships, hope, in-person social connectedness and, to some extent, online social connectedness were associated with fewer symptoms of depression, anxiety and psychotic-like experiences in a young and vulnerable sample. The confirmation of these findings by further studies, particularly those that can test associations and mediating roles over time, would offer valuable further evidence in favour of the social identity approach to health, specifically the multiple identities hypothesis, identity continuity pathway, connection hypothesis and agency hypothesis. Future research should continue to explore the potentially complex and distinct roles of hope, in-person social connection and online social connection in vulnerable young people's mental health difficulties, with greater consideration for the transdiagnostic roles of the number and type of groups and the level of identification with groups.

## Data Availability

The data associated with this study are available from the corresponding author upon reasonable request.

## Footnotes

a The longitudinal data were insufficient, in terms of both sample size and quality, to be included in the analysis (baseline: *n* = 105; ~6-month follow-up: *n* = 46).

b BCa CI, bias-corrected and accelerated confidence interval.

## Supplementary material

For supplementary material accompanying this paper visit http://doi.org/10.1192/bjo.2023.500. click here to view supplementary material
