# Supplementary material for: The mediating role of social connectedness and hope in the relationship between group membership continuity and mental health problems in vulnerable young people
Source: BJPsych Open. 2023 Jul 19;9(4):e130. doi: 10.1192/bjo.2023.500 (PMC10375864; doi:10.1192/bjo.2023.500)
Supplement: Supplementary file 1 [file S2056472423005008sup001.docx]

**Supplementary Information**

**Title:** The mediating role of social connectedness and hope in the relationship between group membership continuity and mental health problems for vulnerable young people

[1. Power Calculations 2](#_Toc128562455)

[2. Data Screening 2](#_Toc128562456)

[3. Mediation Models examining Hypothesis 4 2](#_Toc128562457)

[4. Post-Hoc Exploratory Partial Correlations 5](#_Toc128562458)

[5. References 6](#_Toc128562459)

# **Power Calculations**

Power calculations indicated power of 80% or higher to compute bivariate correlations (minimum N = 94, α = .05, and effect size of .03 using G*Power version 3.1.9.6) and simple mediation models (minimum N = 94, 95% confidence intervals and coefficients [see Table 2 in main paper] using Monte Carlo Power Analysis for Indirect Effects^1^). Two of the planned mediation models (testing the mediating role of online social connectedness in the relationship between membership continuity and psychotic-like experiences, and the mediating role of hope in the relationship between membership continuity and anxiety) did not achieve sufficient power but were run as they met the recommended sample size to model parameter ratio of 20:1^2^.

# **Data Screening**

Missing data were present in each study variable (see Table 2 in main paper), proportionally ranging from 3.81% to 7.62%. Independent t-tests and chi-square tests of association, corrected for multiple testing, identified no significant patterns of missing data across age, gender, ethnicity or each study variable. Where less than 20% of the individual items comprising a study variable score were missing, person-mean imputation was performed^3^. One extreme univariate outlier (z score > 3.29) was identified in the CAPE-P15 variable. The outlier was maintained as its removal had no significant influence on the overall conclusions. No multivariate outliers were detected (Mahalanobis distance > 13.82) and scatterplots supported linearity of associations. The assumptions of independent errors (Durbin-Watson values close to 2) and multicollinearity (VIF scores < 10) were met. P-P plots revealed non-normally distributed residuals for the models predicting anxiety and psychotic-like experiences. Heteroscedasticity was present for each model predicting psychotic-like experiences. Where applicable, robust confidence intervals and standard errors were computed for all analyses.

# **Mediation Models examining Hypothesis 4**

Path b

*b = -.41, p <.001*

*(β* = -.60)

Path c’

Direct effect: *b* = -.09 (*β* = -.07), *p = .43*

Path a

*b = .72, p <.001*

*(β* = .39)

In-Person Social Connectedness

Membership Continuity

Depression (PHQ-9)

Total effect: *b* = -.38 (*β* = -.30), *p = .003*

Indirect effect: *b* = -.28 (*β* = -.23), *95% BCa CI* ***[-.59, -.07]***

**Figure 1**

*Model of group membership continuity as a predictor of depression, mediated by in-person social connectedness*

Path b

*b = -.31, p <.001*

*(β* = -.46)

Path a

*b = .55, p = .004*

*(β* = .29)

Online Social Connectedness

Membership Continuity

Depression (PHQ-9)

Total effect: *b = -.37 (β* = -.30), *p = .003*

Indirect effect: *b* = -.17 (*β* = -.14), *95% BCa CI* ***[-.40, -.02]***

**Figure 2**

*Model of group membership continuity as a predictor of depression, mediated by online social connectedness*

Path c’

Direct effect: *b* = -.21 (*β* = -.17), *p = .07*

Path b

*b = -.27, p <.001*

*(β* = -.47*)*

Path c’

Direct effect: *b* = -.09 (*β* = -.07), *p = .50*

Path a

*b = 1.02, p <.001*

*(β* = .46)

Hope

Membership Continuity

Depression (PHQ-9)

Total effect: *b* = -.36 (*β* = -.29), *p = .005*

Indirect effect: *b* = -.27 (*β* = -.22), *95% BCa CI* ***[-.50, -.10]***

**Figure 3**

*Model of group membership continuity as a predictor of depression, mediated by hope*

Path b

*b* = -.30, *p* = <.001

(*β* = -.50)

Path c’

Direct effect: *b* = -.16 (*β* = -.14), *p = .13*

Path a

*b* = .69, *p* = <.001

(*β* = .38)

In-Person Social Connectedness

Membership Continuity

Anxiety (GAD-7)

Total effect: *b* = -.37 (*β* = -.33), *p = .001*

Indirect effect: *b* = -.21 (*β* = -.19), *95% BCa CI* ***[-.44, -.05]***

**Figure 4**

*Model of group membership continuity as a predictor of anxiety, mediated by in-person social connectedness*

Path b

*b = -.25, p = <.001*

*(β* = -.42)

Path c’

Direct effect: *b* = -.24 (*β* = -.22), *p = .02*

Path a

*b = .48, p = .01*

*(β* = .26)

Online Social Connectedness

Membership Continuity

Anxiety (GAD-7)

Total effect: *b* = -.36 (*β* = -.33), *p = .001*

Indirect effect: *b* = -.12 (*β* = -.11), *95% BCa CI [-.30, .00]*

**Figure 5**

*Model of group membership continuity as a predictor of anxiety, mediated by online social connectedness*

Path b

*b = -.12, p = .02*

*(β* = -.25)

Path c’

Direct effect: *b* = -.21 (*β* = -.19), *p = .08*

Path a

*b = 1.06, p = <.001*

*(β* = .48)

Hope

Membership Continuity

Anxiety (GAD-7)

Total effect: *b* = -.34 (*β* = -.31), *p = .002*

Indirect effect: *b* = -.13 (*β* = -.12), *95% BCa CI [-.30, .03]*

**Figure 6**

*Model of group membership continuity as a predictor of anxiety, mediated by hope*

Path b

*b = -.29, p = .004*

*(β* = -.31)

Path c’

Direct effect: *b* = -.29 (*β* = -.17), *p = .10*

Path a

*b = .70, p = <.001*

*(β* = .38)

In-Person Social Connectedness

Membership Continuity

Psychotic-like experiences (CAPE-15)

Total effect: *b* = -.49 (*β* = -.28), *p = .005*

Indirect effect: *b -.20 (β* = -.12), *95% BCa CI [-.59, .01]*

**Figure 7**

*Model of group membership continuity as a predictor of psychotic-like experiences, mediated by in-person social connectedness*

Path b

*b = -.24, p = .01*

*(β* = -.25*)*

Path c’

Direct effect: *b* = -.38 (*β* = -.22), *p = .03*

Path a

*b = .48, p = <.01*

*(β* = .26)

Online Social Connectedness

Membership Continuity

Psychotic-like experiences (CAPE-15)

Total effect: *b = -.50 (β* = -.28), *p = .005*

Indirect effect: *b* = -.11 (*β* = -.06), *95% BCa CI [-.42, .03]*

**Figure 8**

*Model of group membership continuity as a predictor of psychotic-like experiences, mediated by online social connectedness*

Path b

*b = -.28, p = .001*

*(β* = -.36)

Path c’

Direct effect: *b* = -.21 (*β* = -.12), *p = .27*

Path a

*b = 1.02, p = <.001*

*(β* = .46)

Hope

Membership Continuity

Psychotic-like experiences (CAPE-15)

Total effect: *b* = -.50 (*β* = -.28), *p = .005*

Indirect effect: *b* = -.29 (*β* = -.16), *95% BCa CI* ***[-.58, -.07]***

**Figure 9**

*Model of group membership continuity as a predictor of psychotic-like experiences, mediated by hope*

# **Post-Hoc Exploratory Partial Correlations**

Exploratory partial correlations were run to investigate the unique relationships between online social connectedness and in-person social connectedness with self-rated mental health problems (depression, anxiety and psychotic-like experiences). Table S1 shows that when controlling for online social connectedness, the relationships between in-person social connectedness and each self-rated mental health problem remained significant, explaining 19.36% of the remaining shared variance with symptoms of depression, 11.56% of the remaining shared variance with symptoms of anxiety, and 4.84% of the remaining shared variance with symptoms of psychotic-like experiences. When controlling for in-person social connectedness, the relationships between online social connectedness and each self-rated mental health problem were no longer significant, explaining 0.03% of the remaining shared variance with symptoms of depression, 1.2% of the remaining shared variance with symptoms of anxiety, and 0.02% of the remaining shared variance with symptoms of psychotic-like experiences. This suggests an agreement with the proposition that when considering associations with mental health problems, a sense of connection and belonging from in-person interactions and relationships may play a more pertinent role than a sense of connection from online interactions and relationships.

| **Table S1**  ***Summary statistics for exploratory partial correlations between in-person social connectedness, online social connectedness, and self-rated mental health problems*** | | | | | | | | | | |
| --- | --- | --- | --- | --- | --- | --- | --- | --- | --- | --- |
| **Variable** | **In-Person Social Connectedness**^†^ | | | | | **Online Social Connectedness**^‡^ | | | | |
|  | ***r*** | ***p*** | ***df*** | **95% BCa CI** | **SE** | ***r*** | ***p*** | ***df*** | **95% BCa CI** | **SE** |
| Depression (PHQ-9) | -.44 | <.001 | 94 | [-.60, -.27] | .09 | -.05 | .62 | 94 | [-.25, .12] | .10 |
| Anxiety (GAD-7) | -.34 | <.001 | 95 | [-.52, -.14] | .10 | -.11 | .29 | 95 | [-.30, .10] | .10 |
| Psychotic-like experiences (CAPE-15) | -.22 | .03 | 93 | [-.39, -.07] | .09 | -.04 | .68 | 93 | [-.22, .11] | .09 |
| *Note.* Bias controlled and accelerated confidence intervals based on 5000 bootstrap samples  ^†^ Partial correlations controlling for online social connectedness; ^‡^ Partial correlations controlling for in-person social connectedness | | | | | | | | | | |

# **References**

[1] Schoemann AM, Boulton AJ, Short SD. Determining power and sample size for simple and complex mediation models. *Social Psychological and Personality Science* 2017; **8**(4): 379-386.

[2] Kline RB. *Principles and Practice of Structural Equation Modeling* 4^th^ ed. London: Guilford Press, 2016

[3] Siver S. *Methods for Handling Missing Data for Multiple-Item Questionnaires* [conference paper]. River Cities I/O Conference, Chattanooga, TN, 2016
